# Developing symptom clusters: linking inflammatory biomarkers to depressive symptom profiles

**DOI:** 10.1038/s41398-022-01900-6

**Published:** 2022-03-31

**Authors:** Sabina I. Franklyn, Jayme Stewart, Cecile Beaurepaire, Emily Thaw, Robyn J. McQuaid

**Affiliations:** 1 Department of Psychology, Carleton University, Ottawa, ON Canada; 2 University of Ottawa Institute of Mental Health Research, Ottawa, ON Canada; 3 Department of Neuroscience, Carleton University, Ottawa, ON Canada

**Keywords:** Predictive markers, Depression

## Abstract

Considering the burden of depression and the lack of efficacy of available treatments, there is a need for biomarkers to predict tailored or personalized treatments. However, identifying reliable biomarkers for depression has been challenging, likely owing to the vast symptom heterogeneity and high rates of comorbidity that exists. Examining biomarkers that map onto dimensions of depression as well as shared symptoms/constructs that cut across disorders could be most effective for informing personalized treatment approaches. With a sample of 539 young adults, we conducted a principal component analysis (PCA) followed by hierarchical cluster analysis to develop transdiagnostic clusters of depression and anxiety symptoms. We collected blood to assess whether neuroendocrine (cortisol) and inflammatory profiles (C-reactive protein (CRP), Interleukin (IL)-6, and tumor necrosis factor (TNF) – α) could be used to differentiate symptom clusters. Six distinct clusters were identified that differed significantly on symptom dimensions including somatic anxiety, general anxiety, anhedonia, and neurovegetative depression. Moreover, the neurovegetative depression cluster displayed significantly elevated CRP levels compared to other clusters. In fact, inflammation was not strongly associated with overall depression scores or severity, but rather related to specific features of depression marked by eating, appetite, and tiredness. This study emphasizes the importance of characterizing the biological underpinnings of symptom dimensions and subtypes to better understand the etiology of complex mental health disorders such as depression.

## Introduction

Individuals within DSM-5 categories can display significant heterogeneity in symptom presentations [1]. Additionally, high rates of comorbidities occur, such that ~50% of individuals with major depressive disorders (MDD) also experience an anxiety disorder [2]. In fact, constructs relating to depressive symptoms such as anhedonia and altered cognitive functioning cut across diagnostic boundaries [3, 4]. Together, the variability in symptom profiles and comorbid features across disorders can impede treatment efficacy [5]. With approximately only one-third of individuals receiving an antidepressant treatment reaching remission [6], a better understanding of the neurobiology of complex mental health disorders is needed, which reflects the heterogeneity and comorbidity that exists [7, 8]. To address this need, the Research Domain Criteria (RDoC) introduced by the US National Institute of Mental Health, is focused on shifting biomarker research away from the constraints of diagnostic categories in an effort to identify the neurobiology of dimensions that cut across disorders [9].

Individuals with depression frequently exhibit elevated peripheral levels of cortisol and inflammatory markers relative to non-depressed individuals [10, 11]. Specifically, the inflammatory marker C-reactive protein (CRP) and pro-inflammatory cytokines, interleukin (IL)-6 and tumor necrosis factor (TNF)-α, which are often elevated in MDD [12], are thought to be central in the development, prognosis, and maintenance of depressive symptoms [13]. However, not all individuals with depression display increased cortisol and inflammatory factors, suggesting that these markers may only be observed in a subset of depressed individuals who display specific symptomatologies [14, 15]. In fact, associations between inflammation and specific depressive symptom, including concentration and anhedonia, failed to remain significant when considering neurovegetative symptoms in the analyses [14]. In line with these data, interferon (IFN)-α therapy, a cytokine used to treat certain cancers, resulted in the development of neurovegetative and somatic symptoms within 2 weeks, which were less responsive to antidepressant treatment compared to symptoms of depressed mood that occurred much later [16]. These differences in the timing of symptoms and treatment response suggest that different biological mechanisms underlie depressive dimensions [16].

There is evidence that shifting biomarker research away from the constraints of diagnostic categories can effectively differentiate dimensions that cut across disorders according to neurobiology, which has implications for informing personalized or tailored treatments [8, 9, 17]. Thus, the current investigation focused on characterizing meaningful subtypes (i.e., clusters) of individuals according to depressive and anxiety symptomatologies, and explored whether peripheral biomarkers, including cortisol, CRP, IL-6, and TNF-α levels could be used to differentiate symptom clusters. We predicted that distinct symptom-derived subtypes could be identified and that these subtypes would map onto neuroendocrine and inflammatory biomarkers. More specifically, our a priori hypothesis was that the subtype(s) with the greatest neurovegetative depressive features would be marked by the highest inflammatory profiles.

## Methods and materials

### Participants

Participants comprised 539 undergraduate students ranging from 17–29 years of age (Mean _age_ = 19.38; SD = 2.15). Of participants, 76.3% identified as women (*n* = 411), 23.2% as men (*n* = 125), and 0.6% as gender non-conforming (*n* = 3). Participants reported diverse ethnic backgrounds, including White (59.4%; *n* = 320), Black (10.9%; *n* = 59), Arab/West Asian (7.6%; *n* = 41), Asian (6.1%; *n* = 33), South Asian (5.8%; *n* = 31), Latin American/Hispanic (2.6%, *n* = 14), South East Asian (1.9%, *n* = 10), and Indigenous (0.6%, *n* = 3), with 10.1% reporting their ethnicity as other (i.e., mixed ethnicities; *n* = 54).

Over one-third of participants (35.1%; *n* = 189) self-reported having a current mental health condition. Of those who reported a current mental health condition, 42.3% (*n* = 80) reported anxiety, 30.7% (*n* = 58) reported comorbid anxiety and depression, 16.9% (*n* = 32) depression, and 7.9% (*n* = 15) reported “other”. Moreover, 20% (*n* = 108) of participants reported currently receiving treatment for a mental health condition. Of those who reported receiving mental health treatment, 25.5% (*n* = 27) reported using anti-depressants, 24.5% (*n* = 26) reported attending therapy, 18.9% (*n* = 20) reported using a combination of therapy and medication, 0.6% (*n* = 3) reported using anxiolytics, and 28.3% (*n* = 30) reported “other”.

### Procedure

Participants were recruited via the university’s online research system. Participants were considered eligible if they were between the ages of 17–29 years, were fluent in English. All participants provided informed consent, following this, participants were given a questionnaire booklet assessing detailed demographic and medical health information (e.g., current and past physical and mental health disorders, height, weight, exercise, etc.), as well as current mood including anxiety and depressive symptoms. Once completed, participants were screened for willingness and eligibility to provide a blood sample. Participants who were willing and eligible to provide a blood sample (i.e., did not have any auto-immune disorders, were not taking any anti-inflammatory medication, and had never had any blood drawn complications), were provided with a consent form for blood collection. Of participants, 261 provided a blood sample for inflammatory and neuroendocrine assays. No differences existed between participants who either provided or did not provide a blood sample on any study measures (see supplementary materials for detailed statistics). Upon signing the additional consent, a registered phlebotomist collected 5 mL of venous blood into chilled EDTA coated tubes. Once completed, participants were debriefed and provided with course credit. Ethical approval for all procedures was obtained from the Carleton University Research Ethics Board (REB), the Carleton University Biohazard Committee, and the Royal Ottawa Mental Health Centre’s REB.

### Measures

#### Depressive symptoms

The 21-item version of the Beck Depression Inventory (BDI; [18]) was used to assess current depressive symptoms. All items were scored on a scale ranging from 0 (“low”) to 3 (“high”). Moreover, five additional items assessing atypical depressive symptoms, including increased sleep, fatigue, eating, and changes in diet were added to the BDI. These items were subsequently incorporated into a principal component analysis (PCA; for details see supplementary materials). In addition to being used on an item-level to determine symptom clusters, BDI items were summed to assess relationships (*α* = 0.89).

#### Anxiety symptoms

The 21-item version of the Beck Anxiety Inventory (BAI; [19]) was used to assess current symptoms of anxiety. All 21 items were scored from 0 (“not having experienced that symptom”) to 3 (“experiencing that symptom frequently”). Items in this scale were inputted into the PCA to determine symptom dimensions in addition to being summed in order to examine a total anxiety score (*α* = 0.90).

#### Depression anxiety stress scale

The Depression, Anxiety and Stress Scale, version 21 (DASS-21; [20]) is a 21-item self-report questionnaire used to assess mood, anxiety, and stress symptoms. Participants responded to each item using a four-point Likert scale ranging from 0 (“did not apply to me at all”) to 3 (“applied to me very much”). All items were inputted into the PCA. The DASS comprises three subscales. The depression subscale (*α* = 0.87) assesses negative emotional states associated with depression (e.g., dysphoria, hopelessness, devaluation of life, self-deprecation, lack of interest/involvement, and anhedonia). The anxiety subscale (*α* = 0.79) assesses negative emotional states associated with anxiety (e.g., autonomic arousal, skeletal muscle effects, situational anxiety, and the subjective experience of anxious affect). The stress subscale (*α* = 0.81) assesses negative emotional states associated with stress by asking questions regarding levels of chronic non-specific arousal (e.g., difficulty relaxing, nervous arousal, impatience, irritability, and agitation).

#### Anhedonia

Anhedonia, a feature of depression that is characterized by a lack of pleasure, was measured using the Snaith-Hamilton Pleasure Scale (SHAPS; [21]). The SHAPS assesses an individual’s current ability to experience pleasure. The SHAPS includes 14 items that describe pleasurable experiences (e.g., “I would enjoy being with family or close friends”), where items are scored on a scale from 1 (“strongly agree”) to 4 (“strongly disagree”). Responses to items in this scale were summed to represent a total anhedonia score (*α* = 0.90).

#### Blood collection

Laboratory sessions were held between 1200 and 1530 h to limit hormonal diurnal variations. Study session time did not map onto altered cortisol levels (see supplemental section). Blood samples were collected after the completion of the questionnaires based on eligibility. Venous blood samples (5 mL) from the antecubital fossa were collected directly into chilled EDTA coated vacutainer tubes by a registered phlebotomist, immediately placed on ice, and centrifuged for 20 min at 4 °C and 1000 g. Plasma was then aliquoted into microtubes and frozen at −80 °C until required for cortisol and inflammatory assays.

#### Plasma inflammatory assays

Circulating levels of CRP, IL-6, and TNF-α were determined in duplicate by high sensitivity human ELISA kits. The CRP kits were obtained from Life Technologies (Fisher Scientific; Catalog #: LSKHA0031) and IL-6 (Catalog #: HS600C) and TNF-α kits (Catalog #: SSTA00E) were obtained from R&D systems (Bio-Techne Canada). The assays were performed according to the manufacturer’s instructions. The inter- and intra-assay variability was less than 15%.

#### Plasma cortisol assay

Plasma cortisol was determined in duplicate by radioimmunoassay (RIA) using a Cortisol Coated Tube RIA kit obtained from MP Biomedicals, LLC (Catalog #: 07-221105R). The assay was performed according to the manufacturer’s instructions. The inter- and intra-assay variability was less than 10% and the minimum detectable concentration was 0.17 μg/dL.

### Statistical analyses

All statistical analyses were conducted using IMB SPSS Statistics, version 27, significance was considered at *p* < 0.05 (two-sided). Detailed data screening and transformation information are provided in the supplemental materials. Pearson correlations assessed relationships between total BDI, BAI, and Anhedonia scores with biomarkers. For the data-driven clustering approach, a principal components analysis (PCA) was conducted on the 68 combined items from the BDI, BAI, and DASS to reduce the number of items into a more manageable size while maintaining variance. The sample size was determined based on the recommendations for PCA analyses (i.e., 500 cases is considered very good; [22]). The number of components retained was determined based on sample size, the scree plot, and the percentage of variance explained using Kaiser’s criterion (see supplementary materials). The component scores of the four PCA components were then input into a hierarchical agglomerative cluster analysis using the squared Euclidian distance metric and Ward’s method to identify groups of individuals with similar symptomatologies. The number of clusters was determined using the agglomeration schedule and resulting dendrogram (see supplementary materials). Crosstabs were examined to describe symptom clusters according to categorical variables (i.e., gender, mental health-related variables), and when appropriate chi-square tests were conducted to assess differences. Finally, analyses of variance (ANOVAs) were performed to examine differences between groups/clusters on continuous or scaled variables including, BDI, BAI, anhedonia, and peripheral biomarkers. As *N* = 261 participants provided blood samples, additional power analyses using G*Power3 were performed for ANOVAs assessing biomarkers, and confirmed sufficient power, ranging from 0.80 to 0.89, depending on the specific biomarker assessed [23]. Planned follow-up comparisons were made in relation to the healthy control cluster and the cluster of interest for that outcome (nine comparisons/outcome), thus, a Bonferroni correction was applied, and effects were considered significant at the corrected *p* value (*p* < 0.006). Analyses of covariance (ANCOVAs) were also performed to control for BMI when assessing inflammatory markers, for these analyses, the sample size is reduced due to missing BMI data. Similarly, when assessing inflammatory markers, ANCOVAs were performed controlling for treatment for mental health disorders and for ongoing infections, (e.g., having a cold) and the results remained unchanged.

## Results

### Correlations

A bivariate correlation table including the associations between depressive, anxiety, and anhedonia symptoms with all biomarkers can be found in Supplementary Table 1.

### Principal component analysis

A principal components analysis (PCA) was conducted on the 68 combined items from the BDI, BAI, and DASS using an oblique rotation (promax). The Kaiser–Meyer–Olkin (KMO) index of sampling adequacy was excellent (KMO = 0.93) with all individual item KMO values exceeding 0.65 indicating that PCA was appropriate [24]. An initial analysis was run to obtain eigenvalues for each dimension and to determine the appropriate number of components to retain. Fourteen components had eigenvalues over Kaiser’s criteria of 1, whereas the scree plot was ambiguous and showed inflections supporting either a 2 or 4 component structure. Accounting for a cumulative 39.65% of the variance, the four-component structure was retained. Based on component loadings, the four dimensions were named anhedonia, somatic anxiety, general anxiety, and neurovegetative depression (for detailed loadings and dimension results, see Supplementary Table 2).

### Cluster analysis and characterization

**Fig. 1:**
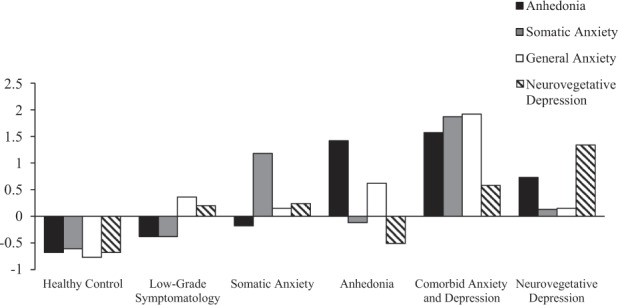
Symptom cluster solution among mean standardized PCA dimension scores. This comprised six clusters including healthy control, low-grade symptomatology, somatic anxiety, anhedonia, comorbid anxiety and depression, and a neurovegetative depression cluster.

The agglomerative hierarchical cluster analysis identified a six-cluster (or subtype) solution (Supplementary Table 3 and Supplementary Fig. 1). As shown in Fig. 1, these clusters included: healthy control (*n* = 230; 42.7%), low-grade symptomatology (*n* = 83; 15.4%), somatic anxiety (*n* = 52; 9.6%), anhedonia (*n* = 35; 6.5%), comorbid anxiety and depression (*n* = 55; 10.2%), and neurovegetative depression (*n* = 84; 15.6%). Each cluster had a distinct symptom profile that differed significantly on the four PCA dimensions (Table 1).

**Table 1:** Distinction between clusters according to symptom component mean scores.

	Type cluster, Mean *z*-score (SE)						Test of difference	
PCA dimensions	Healthy control	Low-grade symptomatology	Somatic anxiety	Anhedonia	Comorbid anxiety and depression	Neurovegetative depression	*F* (5,533)	*p* value
Anhedonia	−0.68 (0.03)	−0.38 (0.04)	−0.18 (0.06)	1.42 (0.10)	1.57 (0.12)	0.73 (0.07)	233.58	<0.001
Somatic Anxiety	−0.61 (0.03)	−0.38 (0.05)	1.18 (0.08)	−0.12 (0.11)	1.87 (0.13)	0.13 (0.06)	222.66	<0.001
General Anxiety	−0.77 (0.03)	0.36 (0.06)	0.15 (0.08)	0.62 (0.11)	1.92 (0.08)	0.15 (0.09)	231.81	<0.001
Neurovegetative	−0.68 (0.04)	0.20 (0.07)	0.24 (0.10)	−0.51 (0.11)	0.58 (0.13)	1.34 (0.09)	124.58	<0.001

*SE* standard error.

In addition to determining how cluster membership differed based on the PCA components, it was of interest to characterize the clusters against other relevant descriptive and mental health variables. As seen in Table 2, clusters differed according to gender, χ^2^(5, *N* = 536) = 38.661, *p* < 0.001. In this regard, approximately two-thirds of men fell within the healthy control cluster, whereas this was only the case for just over one-third of women (36.3%). As expected, participants who self-reported having a current mental health condition differed according to cluster membership, χ^2^(5, *N* = 534) = 112.991, *p* < 0.001. Specifically, the majority of those that did not report having a mental health condition fell within the healthy control cluster (see Table 2). Descriptive statistics regarding the type of mental health condition reported according to the cluster is displayed in Table 2.

**Table 2:** Characteristics of clusters according to gender and mental health-related factors.

	Gender *n* (%)		Current mental health condition *n* (%)		Self-reported diagnosis *n* (%)			Currently receiving mental health treatment *n* (%)	
	Women *n* = 411	Men *n* = 125	Yes *n* = 189	No *n* = 345	Depression *n* = 32	Anxiety *n* = 80	Depression and anxiety *n* = 58	Yes *n* = 108	No *n* = 80
Healthy control	149 (36.3)	81 (64.8)	31 (16.4)	197 (57.1)	7 (21.9)	13 (16.3)	4 (1.8)	16 (14.8)	13 (16.3)
Low-grade symptomatology	68 (16.5)	15 (12.0)	32 (16.9)	50 (14.5)	3 (9.4)	19 (23.8)	8 (13.8)	13 (12.0)	20 (25.0)
Somatic anxiety	47 (11.4)	5 (4.0)	23 (12.2)	29 (8.4)	2 (6.3)	16 (20.00)	3 (5.2)	13 (12.0)	10 (12.5)
Anhedonia	25 (6.1)	8 (6.4)	16 (8.5)	19 (5.5)	7 (21.9)	3 (3.8)	4 (6.9)	8 (7.4)	6 (7.5)
Comorbid anxiety and depression	52 (12.7)	2 (1.6)	44 (23.3)	9 (2.6)	2 (3.6)	17 (21.3)	23 (39.7)	32 (29.6)	12 (15.0)
Neurovegetative depression	70 (17.0)	14 (11.2)	43 (22.8)	41 (11.9)	11 (34.4)	12 (15.0)	16 (27.6)	26 (24.1)	19 (23.8)

### Clusters and mood scores

**Fig. 2:**
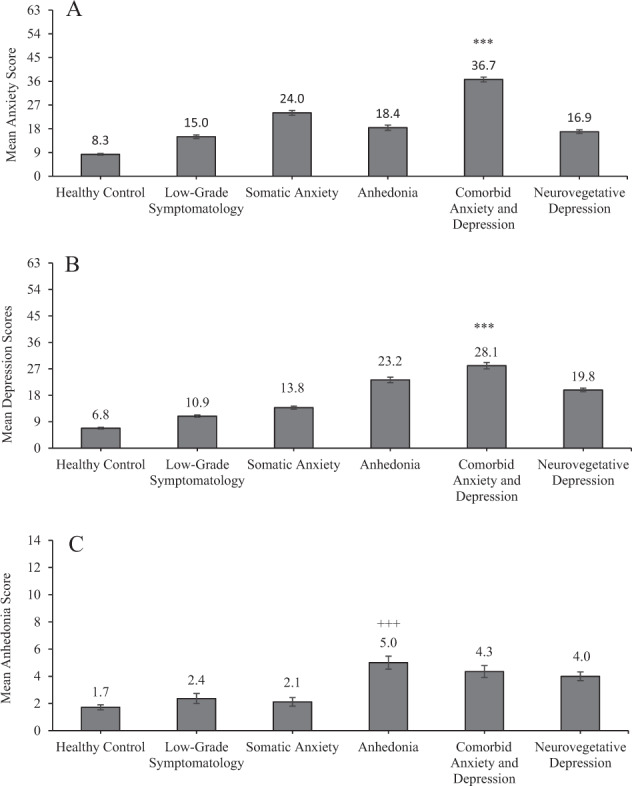
Anxiety, depression, and anhedonia scores according to symptom cluster (mean ± standard error). ***p < 0.001 compared to all other groups for anxiety (**A**) and depressive (**B**) symptoms, and ^+++^*p* < 0.001 compared to healthy control, low-grade symptomatology, and somatic anxiety clusters for anhedonia symptoms (**C**).

It was of interest to confirm that the clusters scoring highest on the mood and anxiety PCA loadings would also display the highest total BAI and BDI scores. As expected, anxiety and depressive scores significantly differed according to cluster membership, *F*(5,533) = 241.73, *p* < 0.001, η^2^ = 0.69 and *F*(5,533) = 224.83, *p* < 0.001, η^2^ = 0.68, respectively. Indeed, as indicated in Fig. 2, and confirmed by follow-up comparisons, anxiety, and depressive scores were highest in the comorbid anxiety and depression cluster, which differed from all other clusters (*p’*s < 0.001). As predicted, all symptom cluster groups exhibited higher anxiety and depressive scores compared to healthy controls (*p’*s < 0.001; Fig. 2A, B).

A one-way ANOVA confirmed that anhedonia scores differed according to cluster membership, *F*(5,533) = 16.79, *p* < 0.001, η^2^ = 0.14. Comparisons revealed that the anhedonia cluster had higher mean anhedonia scores compared to the healthy control, *p* < 0.001, low-grade symptomatology, *p* < 0.001, and somatic anxiety clusters, *p* < 0.001 (Fig. 2C). However, the anhedonia cluster did not differ relative to the comorbid anxiety and depression, *p* = 0.91, or the neurovegetative clusters, *p* = 0.54, in which depression is a major component. Not unexpectedly and as shown in Fig. 2C, only the anhedonia, *p* < 0.001, comorbid anxiety and depression, *p* < 0.001, and neurovegetative, *p* < 0.001 clusters significantly differed compared to healthy controls.

### Clusters and biological factors

**Fig. 3:**
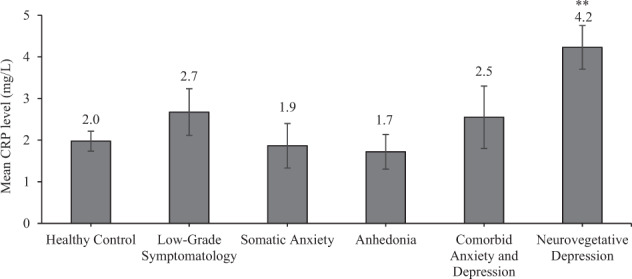
Basal levels of CRP according to symptom cluster (mean ± standard error). **p < 0.01 compared to healthy control, somatic anxiety, and anhedonia clusters.

A series of one-way ANOVAs were conducted to determine how biological factors differed based on cluster membership. The first one-way ANOVA revealed that cortisol did not differ significantly with cluster membership, *F*(5,255) = 1.39, *p* = 0.23, η^2^ = 0.03, whereas the analysis of CRP revealed a significant difference as a function of cluster membership, *F*(5,254) = 4.40, *p* = 0.001, η^2^ = 0.08. Follow-up comparisons revealed that CRP levels in the neurovegetative cluster were significantly greater compared to the healthy controls, *p* < 0.001, anhedonia *p* = 0.003, and somatic anxiety clusters, *p* = 0.001. However, CRP in the neurovegetative cluster was not significantly elevated compared to the low-grade symptomatology, *p* = 0.02, or the comorbid anxiety and depressive, *p* = 0.03, clusters at the Bonferroni corrected cut-off of *p* < 0.006 (Fig. 3). Moreover, while the neurovegetative cluster differed from healthy controls*, p* < 0.001, there were no differences between healthy controls and the other clusters in CRP levels, including low-grade symptomatology, *p* = 0.21, the comorbid anxiety and depression, *p* = 0.40, anhedonia, *p* = 0.74, or somatic anxiety, *p* = 0.86. When controlling for BMI, clusters remained significant with respect to CRP, *F*(5,115) = 2.70, *p* = 0.024, η^2^ = 0.11. In this model, BMI did not differ by cluster, *F*(1,115) = 2.59, *p* = 0.11, η^2^ = 0.02. Similarly, when controlling for treatment type, clusters remained significant regarding CRP, *F*(5,253) = 4.59, *p* = 0.001, η^2^ = 0.08. In this model, treatment type did not differ by cluster, *F*(1,253) = 0.96, *p* = 0.33, η^2^ = 0.004.

In contrast to CRP, clusters did not differ for IL-6, *F*(5,204) = 1.31, *p* = 0.26, η^2^ = 0.03 or TNF-α levels, *F*(5,232) = 0.82, *p* = 0.54, η^2^ = 0.02. However, when controlling for BMI, clusters tended to differ somewhat in relation to IL-6, although, this only approached significance, *F*(5,90) = 2.16, *p* = 0.066, η^2^ = 0.02. The ANCOVA controlling for BMI examining TNF-α levels according to cluster, remained non-significant, *F*(5,103) = 1.21, *p* = 0.31, η^2^ = 0.06. In addition, when controlling for treatment type, both IL-6 and TNF-α remained non-significant with respect to cluster membership, *F*(5,252) = 1.20, *p* = 0.31, η^2^ = 0.02 and *F*(5,231) = 0.77, *p* = 0.58, η^2^ = 0.02, respectively.

## Discussion

The current study identified six distinct symptom clusters/subtypes that differed according to mood states and inflammatory levels. Individuals in these subtypes displayed various combinations of depressive and/or anxiety symptom features, differing across somatic anxiety, general anxiety, anhedonia, and neurovegetative depression dimensions. The six clusters included: *healthy control*, *low-grade symptomatology*, and *somatic anxiety*, which was marked by physical symptoms of anxiety in the absence of other features, as well as *anhedonia*, which was characterized by mood disturbances (e.g., loss of pleasure) in the absence of more physical symptoms (e.g., tired, appetite), *neurovegetative depression*, which displayed predominantly physical features of depression with secondary mood disturbances, and lastly a *comorbid anxiety and depression* cluster, which was characterized by a combination of both somatic anxiety and general anxiety (excessive and constant worrying), together with symptoms of anhedonia.

The *comorbid anxiety and depression* cluster emerged to be an important subtype. In particular, these individuals displayed the highest depression and anxiety scores compared to all other groups, which is consistent with reports that greater symptom severity is present among individuals with comorbid mood and anxiety disorders [25, 26]. Paradoxically, while the comorbid cluster displayed the most pronounced mood disturbances, it did not demonstrate the most extreme or elevated neuroendocrine or inflammatory biomarker profiles. This raises the question as to whether this comorbid mental health cluster might also be marked by physical illness comorbidities, and in this instance, if biomarkers that reflect cardiovascular and/or metabolic measures (other than BMI) might have been elevated in this cluster. Further investigations using a transdiagnostic or RDoC approach to examine a wider range of biomarkers associated with comorbid mental and physical symptom subtypes, perhaps including an allostatic load composite measure, could be important. Regardless, the current data indicate that assessing total scores or overall symptom severity in relation to neuroendocrine and inflammatory biomarkers might not be particularly informative; rather specific symptoms/subtypes were most closely tied to biomarkers.

The *neurovegetative* subtype or cluster of individuals was characterized by anhedonia together with physical symptoms of depression including increased appetite, weight gain, and tiredness, which is in-line with an atypical subtype of neurovegetative depression. This atypical neurovegetative subtype demonstrated significantly elevated circulating CRP levels (i.e., mean scores of 4.2 mg/L), beyond what is considered within a normal or typical range (0–3 mg/L). These comparisons appear to indicate that elevated levels of the inflammatory marker, CRP, are specifically tied to neurovegetative features of depression. In support of this suggestion, associations between CRP and other depressive symptoms, such as cognitive and emotional symptoms (e.g., anhedonia), were no longer significant when controlling for neurovegetative features [14, 27, 28]. Indeed, elevated CRP was previously found among individuals with atypical depression compared to those with melancholic depression [29]. Moreover, the association between inflammatory markers, in particular CRP, has been linked to altered appetite symptoms of depression [30]. While an association between CRP and neurovegetative features is not always found, others reveal that IL-6 levels prospectively predict neurovegetative features [31]. Together these data seem to support an association between neurovegetative features of depression and inflammation. Moreover, our cluster approach allowed comparisons of inflammatory profiles between the atypical neurovegetative subtype to other depressive and/or anxiety subtypes. As neurovegetative symptoms might be more resistant to traditional antidepressant treatments [32, 33], and peripheral CRP levels are elevated among those with treatment-resistant depression [33], these findings together with other reports, could provide important information regarding who might benefit from anti-inflammatory treatments for depression [34].

A relationship existed between basal cortisol levels and overall depression scores, although this was a small effect. Regardless, this is in-line with data indicating that elevated basal cortisol levels are a fairly reliable biomarker of depression [11, 35]. However, it is also suggested that the cortisol-depression link, might depend on the presence of specific depressive symptom profiles [15]. In particular, elevated levels of cortisol have been found in melancholic subtypes of depression, whereas individuals with the atypical neurovegetative subtype appear to have normal or in some cases, low levels of cortisol [15, 36]. In the current study, cortisol levels did not differ according to symptom subtypes, potentially because a melancholic depressive subtype was not derived from the data-driven cluster analysis. Moreover, it is possible that while similar levels of baseline cortisol were displayed across subtypes, differences could have emerged in the context of a stressor or challenge.

### Limitations

There are several limitations associated with the current study. Despite an overall sample size of 539 individuals, one limitation of this study was the relatively small number of individuals in each subtype. Thus, replication of the current findings is needed. Future studies aiming for larger sample sizes will allow for more detailed sub-analyses within clusters, such as detailed sex and gender-based analyses. In the present investigation, there was a much greater ratio of women to men, which is consistent with our previous student studies on mental health [37, 38]. Although depression is more prevalent among women than men [39, 40], including among this young adult age group [41], a more even gender split would have allowed us to discern whether the neurovegetative—inflammation link is more apparent among women or men. Understanding this relationship is particularly important given the suggestion that women are more vulnerable to the depressogenic effects of inflammation when compared to men [42, 43] however, it remains uncertain whether inflammation contributes to the increased rates of depression among females [42].

While the purpose of this study was in-line with an RDoC approach, and thus, was to work outside the constraints of a DSM-V diagnosis and instead focus on symptom dimensions, approximately one-third of this student sample reported a mental health disorder. This was expected as mental health disorders are frequent among university students [44]. However, a student sample might limit generalizability, and therefore, the fact that a handful of other studies have also reported associations between neurovegetative symptomatologies and CRP levels in middle-aged and older adults through different methodologies and approaches is reassuring [27]. Moreover, it would be valuable to assess outcomes among individuals with confirmed DSM-5 diagnoses for input into the cluster analyses. Relatedly, some participants were receiving treatment in the form of various therapies and/or psychopharmacological treatment at the time of this study, which could have potentially normalized levels of pro-inflammatory cytokines, in effect precluding significant findings with IL-6 or TNF-α [45, 46]. However, when controlling for mental health treatment type in the current analyses, our results remained unchanged. Finally, there is a degree of subjectivity when interpreting results of cluster-based analyses, including ensuring that the clusters derived are meaningful. Thus, despite using multiple hallmarks stopping methods for hierarchical clustering, a five-cluster solution could also have been an appropriate alternative to the six-cluster solution retained. Importantly, regardless of the cluster solution in the current study, the link between CRP and the *neurovegetative* subtype persisted.

Overall, the current results suggest that subtypes of anxiety and depressive symptomatologies differ based on the general inflammatory marker CRP. These data may lend themselves to a more in-depth understanding of the neurobiology of specific depressive subtypes, beyond what can be gained from assessing total depressive severity. Moreover, focusing on symptom dimensions, within and across DSM-5 diagnoses, may more accurately reflect the heterogeneity and comorbidity of depression and its underlying etiology.

## Supplementary information

Supplementary Information
