# Correction: Developing symptom clusters: linking inflammatory biomarkers to depressive symptom profiles

**DOI:** 10.1038/s41398-022-01932-y

**Published:** 2022-04-26

**Authors:** Sabina I. Franklyn, Jayme Stewart, Cecile Beaurepaire, Emily Thaw, Robyn J. McQuaid

**Affiliations:** 1 Department of Psychology, Carleton University, Ottawa, ON Canada; 2 University of Ottawa Institute of Mental Health Research, Ottawa, ON Canada; 3 Department of Neuroscience, Carleton University, Ottawa, ON Canada

**Keywords:** Predictive markers, Depression

Correction to: *Translational Psychiatry* 10.1038/s41398-022-01900-6, published online 31 March 2022

The production team removed necessary commas in the reporting of the degrees of freedom for each F-test, significantly changing the meaning of the sample size for each test. This was an error introduced by the publication team. We apologize for these errors. The original article has been corrected.

