# Supplementary material for: Developing symptom clusters: linking inflammatory biomarkers to depressive symptom profiles
Source: Transl Psychiatry. 2022 Mar 31;12:133. doi: 10.1038/s41398-022-01900-6 (PMC8971490; doi:10.1038/s41398-022-01900-6)
Supplement: Supplementary file 1 — Supplementary Information [file 41398_2022_1900_MOESM1_ESM.docx]

**Developing Symptom Clusters: Linking Inflammatory Biomarkers to Depressive Symptom Profiles**

***Supplemental Information***

**Contents**

**Supplemental Text.** Extended Results…………………………………………………………...2

**Supplementary Table S1.** Zero-Order Pearson Correlations between Mood and Biological Measures. ..……………………………..…………………………………………………5

**Supplementary Table S2.** Principal Component Analysis Structure and Loadings of Combined BDI, BAI and DASS Items………………………………………..……………………….6

**Supplementary Table S3.** Percentage Change in Within-Cluster Heterogeneity Based on Number of Clusters Retained by Cluster Analysis………………………………………………....10

**Supplementary Figure S1.** Dendrogram for Hierarchical Clustering…………..…………….....11

**Extended Results**

*Data Screening*

Data were first screened for normality by inspecting histograms, Q-Q plots and P-P plots for each key variable (i.e., BDI, BAI, anhedonia, cortisol, CRP, TNF-α, IL-6). Upon visual inspection, all key variables appeared positively skewed. As such, each variable was transformed. In the case of BDI, BAI, CRP, and TNF-α, a square root transformation was applied to reach normality. Upon visually inspecting the transformed TNF-α variable, three datapoints were identified as potential outliers. As such, the transformed TNF-α variable was next transformed into *z*-scores to examine if any data point fell beyond a *z*-score of ±3.29, which Field (2013) states is indicative of being an extreme outlier. The three cases previously identified fell beyond a *z*-score ±3.29 (i.e., 3.43, 3.57, 6,20) and were removed from subsequent analyses involving TNF-α. For cortisol and IL-6, a logarithmic transformation was performed to reach normality. In the case of anhedonia, transformations did not improve the right skew.

Next, homoscedasticity was assessed using Levene’s test for equality of variance. Levene’s test was significant for BDI (*F*(5, 533) = 13.61, *p* < .001), BAI (*F*(5, 533) = 6.58, *p* < .001), anhedonia (*F*(5, 533) = 2.95, *p* = .012), cortisol (*F*(5, 255) = 2.63, *p* = .024), and CRP (*F*(5, 254) = 2.45, *p* = .035), indicating that the assumption of homogeneity of variances were violated for these variables. Accordingly, for these variables, Welch’s *F* was used as this test corrects for the problem of heteroscedasticity while maintaining power (1).

*ANOVAs with Transformed Variables*

A series of ANOVAs were conducted to determine how clusters were related to transformed mood and biological variables (i.e., BDI. BAI, anhedonia, cortisol, CRP, TNF-α, IL-6). Both depression and anxiety were significantly related to cluster membership (*Welch’s F*(5, 165.38) = 174.45, *p* < .001, η^2^ = .63 and *Welch’s F*(5, 165.17) = 232.64, *p* < .001, η^2^ = .61, respectively). Planned contrasts revealed that depressive and anxiety scores were highest in the comorbid anxiety and depression cluster, which differed from all other clusters (*p’s* < .001). Similarly, anhedonia significantly differed according to cluster membership. *Welch’s F*(5, 155.36) = 16.69, *p* < .001, η^2^ = .14. A series of planned contrasts revealed that the anhedonia cluster had the highest mean anhedonia scores, which were significantly higher compared to the healthy control (*p* < .001), low-grade symptomatology (*p* < .001), and somatic anxiety clusters (*p* < .001). The anhedonia cluster did not significantly differ relative to the comorbid anxiety and depression (*p* = .30) or neurovegetative (*p* = .09) clusters.

In terms of biological markers, CRP was significantly related to cluster membership, *Welch’s F*(5, 70.45) = 3.96, *p* = .003, η^2^ = .08. Planned contrasts revealed that the neurovegetative cluster had the highest mean CRP scores, which was significantly higher than all other clusters (healthy control, *p* < .001; low-grade symptomatology, *p* = .006; comorbid anxiety and depression, *p* = .010; somatic anxiety, *p* = .007; anhedonia, *p* = .001). Alternatively, cortisol, IL-6, and TNF-α were not impacted by group membership (*Welch’s F*(5, 69.65) = 1.30, *p* = .28, η^2^ = .03; *F*(5, 204) = 1.51, *p* = .19, η^2^ = .04; *F*(5, 229) = 0.95, *p* = .45, η^2^ = .02, respectively).

Importantly, this pattern of results was consistent with the results obtained from ANOVAs using the untransformed variables and regular *F*-tests. The only exception to this was that the planned contrasts for the transformed CRP revealed significant differences to all other clusters rather than only to the healthy control (*p* < .001), anhedonia (*p* = .003), and somatic anxiety (*p* = .001) clusters when not transformed. However, the story of the manuscript remains unchanged and, given the potential complications of interpreting transformed variables (2, 3), we have opted to use the untransformed variables in addition to the regular *F*-test in the manuscript.

*Group Differences*

To be sure there were no differences between participants who provided blood samples and those that did not, independent t-tests and chi-square analyses were performed. There were no significant differences between participants who provided a blood sample and those who did not provide a blood sample on BAI total scores (*t*(537) = .80, *p* = .44), BDI total scores (*t*(537) = -.53, *p* = .59), anhedonia (*t*(537) = -.09, *p* = .93), the DASS stress subscale *t*(537) = -.15, *p* = .88), the DASS anxiety subscale (*t*(537) = .63, *p* = .53), the DASS depression subscale (*t*(537) = -.71, *p* = .48), age (*t*(526) = -54, *p* = .59) or gender χ^2^= 2.76, *p* = .1.

To ensure cortisol fluctuations did not differ according to study session time (either at 12pm or 2pm), an independent t-test was run. No significant cortisol differences occurred due to study session time, *t*(259), *p* = .13.

*Bivariate Correlations*

Bivariate correlations (**Supplementary Table S1)** revealed that cortisol had a weak positive relation with depression (*r =* .14; *p* = .028) and anxiety symptoms (*r =* .18; *p* = .004). CRP tended to relate to depressive symptoms, although this only approached significance, *r =* .12, *p* = .052. Alternatively, IL-6 and TNF-α did not relate to depression, anxiety or anhedonia symptoms, however, they did relate to each other, (*r =* .25; *p* < .01) and to CRP, (*r =* .49; *p* < .01), and (*r =* .23; *p* < .01), respectively. Moreover, though cortisol was unrelated to TNF-α, it did demonstrate an inverse relationship with IL-6 (*r* = -.14, *p* = .042), and a positive association with CRP (*r* = .21, *p* = 001).

*Principal Component Analysis*

A principal components analysis (PCA) was conducted on the 68 combined items from the BDI, BAI, and DASS using an oblique rotation (promax). The Kaiser-Meyer-Olkin (KMO) of sampling adequacy was excellent (KMO = .93) with all individual item KMO values exceeding .65 indicating that factor analysis is appropriate (1). An initial analysis was run to obtain eigenvalues for each factor in the data and to determine the appropriate number of components to retain. Fourteen components had eigenvalues over Kaiser’s criteria of 1, whereas the scree plot was ambiguous and showed inflexions supporting either a 2 or 4 component structure. The 4-component structure, accounting for 39.65% of variance, was retained.

Of the 68 items, nine did not load onto any component. These unloaded items comprised one BAI (“fear of dying”), five BDI (“I wake up early every day and can’t get more than 5 hours of sleep”, “I have no appetite at all anymore”, “I am completely absorbed in what I feel/ am concerned about my health”, “I have lost interest in sex completely”, “I have had at least 2-hour increase in sleep length”) and three DASS (“I found myself getting agitated”, “I was intolerant of anything that kept me from getting on with what I was doing”, “I felt that I was rather touchy”) items. PCA factor structure and loadings can be found in **Supplementary Table S2**.

*Agglomerative Hierarchical Clustering*

Agglomerative hierarchical clustering using Ward’s method and the squared Euclidian distance metric was used to classify respondents according to various symptoms of depression, anxiety and stress. To determine the number of clusters in the final solution, both the proportionate increase in heterogeneity to the next stage (as assessed according to the agglomeration schedule; **Supplementary Table S3**) and dendrogram (**Supplementary Figure S1**) were examined. Because we were interested in examining the overlapping nature of depression and anxiety, and given that our PCA identified four unique components, it was decided that a manageable number of clusters from a strategic perspective would be more than four, but less than 10 clusters.

With regards to percentage changes in heterogeneity large increases in heterogeneity from one stage to the next indicate that two relatively dissimilar clusters were joined together, where heterogeneity invariably increases as the number of clusters approach one. Within the bounds previously identified (i.e., between 5 to 9 clusters), and as noted in **Supplementary Table S3**, the largest increase in heterogeneity first occurs when moving from a six- to five-cluster solution (10.51%) indicating that the six-cluster solution has proportionately more within cluster homogeneity than the five-cluster solution. Thus, the six-cluster solution was retained.

The dendrogram is a visual representation of the clustering process, demonstrating the similarly between cases. Inspection of the dendrogram (**Supplementary Figure S1**) further corroborated that a six-cluster solution fits the data.

*Inflammatory Assays*

High sensitivity ELISA assays were used for both TNF-α and IL-6 (R & D Systems). For both cytokines, the assay range was 0.2 -10 pg/mL. The assay sensitivity was 0.049 pg/mL for TNF-α and 0.09 pg/mL for IL-6. As per the manufacturer’s recommendations, samples were not diluted. For TNF-α, only one sample (of 255) had levels below detection and therefore were not included in the final data, whereas for IL-6, eight samples (of 237) had levels below detection. Moreover, samples, with inter- or intra-assay variability values greater than 15% were excluded from analyses.

**Supplementary References**

1. Field A (2013): *Discovering statistics using IBM SPSS Statistics: and sex and drugs and rock ‘n’ roll,* 4th edition*.* Thousands Oak, California: Sage Publications Ltd.
2. Kyu Lee, D. (2020). Data transformation: A focus on the interpretation. *Korean Journal of Anesthesiology, 73*(6), 503-508. <https://doi.org/10.4097/kja.20137>
3. Osborne, J. (2002). Notes on the use of data transformation. *Practical Assessment, Research, and Evaluation, 8*(6), 1-7. https://doi.org/ 10.7275/4vng-5608

**Supplementary Table S1. Zero-Order Pearson Correlations between Mood and Biological Measures**

|  | 1 | 2 | 3 | 4 | 5 | 6 |
| --- | --- | --- | --- | --- | --- | --- |
| 1. Depressive symptoms | -- |  |  |  |  |  |
| 2. Anxiety symptoms | .64** | -- |  |  |  |  |
| 3. Anhedonia | .36** | .19** | -- |  |  |  |
| 4. Cortisol | .14* | .18** | -.05 | -- |  |  |
| 5. CRP | .12^+^ | .04 | -.07 | .21** | -- |  |
| 6. IL-6 | .01 | -.01 | -.06 | -.14* | .49** | -- |
| 7. TNF-α | .10 | .02 | .03 | .00 | .23** | .25** |

*Note.* ^+^*p = .052, *p* < .05. *** p* < .01.

**Supplementary Table S2. Principal Component Analysis** **Structure and Loadings of Combined BDI, BAI and DASS Items**

| Items | Dimension loading | | | |
| --- | --- | --- | --- | --- |
|  | 1 | 2 | 3 | 4 |
| **Dimension 1: Anhedonia** |  |  |  |  |
| DASS21: I felt that life was meaningless (D)^a^ | **0.85** |  |  |  |
| DASS10: I felt that I had nothing to look forward to (D) | **0.79** |  |  |  |
| BDI2: I feel that the future is hopeless and things cannot improve | **0.78** |  |  |  |
| DASS17: I felt I wasn't worth much as a person (D) | **0.77** |  |  |  |
| BDI3: I feel I am a complete failure as a person | **0.74** |  |  |  |
| BDI1: I am so sad or unhappy that I can’t stand it | **0.71** |  |  |  |
| BDI7: I hate myself‎/disgusted‎/disappointed with self | **0.71** |  |  |  |
| BDI9: Suicide Ideation | **0.68** |  |  |  |
| DASS13: I felt down-hearted and blue (D) | **0.66** |  |  |  |
| DASS3: I couldn't seem to experience any positive feeling at all (D) | **0.64** |  |  |  |
| BDI4: I am dissatisfied with everything | **0.63** |  |  |  |
| DASS16: I was unable to become enthusiastic about anything (D) | **0.62** |  |  |  |
| BDI15: I can’t do any work at all‎/productivity | **0.61** |  |  |  |
| BDI5: I feel as though I am very bad or worthless‎/unworthy‎/guilty | **0.55** |  |  |  |
| DASS5: I found it difficult to work up the initiative to do things (D) | **0.55** |  |  |  |
| BDI8: I feel I have many bad faults‎/critical of myself‎/blame myself | **0.53** |  |  |  |
| BDI14: I feel that I am ugly or repulsive looking | **0.42** |  |  |  |
| Items | Dimension loading | | | |
|  | 1 | 2 | 3 | 4 |
| BDI12: I have lost all my interest in other people and don’t care about them at all | **0.42** |  |  |  |
| BDI13: I can’t make decisions at all anymore | **0.41** |  |  |  |
| BDI11: I am irritable all the time | **0.39** |  |  |  |
| BDI6: I feel something bad may happen to me‎/I feel I am being punished‎/I deserve to be | **0.38** |  |  |  |
| BDI10: Uncontrollable crying‎/can’t cry even though want to | **0.38** |  |  |  |
| BDI23: I feel more fatigued than usual lately, and it significantly interferes with my daily functioning | **0.37** |  |  |  |
| **Dimension 2: Somatic Anxiety** |  |  |  |  |
| BAI19: Faint |  | **0.69** |  |  |
| DASS4: I experienced breathing difficulty (e.g., excessively rapid breathing, breathlessness in the absence of physical exertion) (A) | | **0.69** |  |  |
| BAI12: Hands trembling |  | **0.69** |  |  |
| BAI13: Shaky |  | **0.68** |  |  |
| DASS7: I experienced trembling (e.g., in the hands) (A) | | **0.66** |  |  |
| BAI6: Dizzy or light-headed |  | **0.66** |  |  |
| BAI3: Wobbliness in legs |  | **0.66** |  |  |
| BAI15: Difficulty breathing |  | **0.62** |  |  |
| BAI1: Numbness or tingling |  | **0.60** |  |  |
| BAI21: Sweating (not due to heat) |  | **0.52** |  |  |
| BAI8: Unsteady |  | **0.52** |  |  |
| Items | Dimension loading | | | |
|  | 1 | 2 | 3 | 4 |
| BAI11: Feelings of choking |  | **0.51** |  |  |
| BAI2: Feeling hot |  | **0.51** |  |  |
| DASS19: I was aware of the action of my heart in the absence of physical exertion (e.g., sense of heart rate increase, heart missing a beat) (A) | | **0.49** |  |  |
| BAI20: Face flushed |  | **0.46** |  |  |
| BAI18: Indigestion or discomfort in abdomen |  | **0.45** |  |  |
| DASS2: I was aware of dryness in my mouth (A) |  | **0.36** |  |  |
| **Dimension 3: General Anxiety** |  |  |  |  |
| BAI5: Fear of the worst happening |  |  | **0.80** |  |
| DASS20: I felt scared without any good reason (A) |  |  | **0.75** |  |
| BAI17: Scared |  |  | **0.75** |  |
| BAI10: Nervous |  |  | **0.75** |  |
| BAI9: Terrified |  |  | **0.71** |  |
| DASS8: I felt that I was using a lot of nervous energy (S) | |  | **0.67** |  |
| DASS12: I found it difficult to relax (S) |  |  | **0.67** |  |
| BAI14: Fear of losing control |  |  | **0.65** |  |
| DASS15: I felt I was close to panic (A) |  |  | **0.64** |  |
| BAI4: Unable to relax |  |  | **0.61** |  |
| DASS9: I was worried about situations in which I might panic and make a fool of myself (A) | | | **0.61** |  |
| DASS1: I found it hard to wind down (S) |  |  | **0.57** |  |
| Items | Dimension loading | | | |
|  | 1 | 2 | 3 | 4 |
| BAI7: Heart pounding or racing |  | 0.37 | **0.48** |  |
| DASS6: I tended to over-react to situations (S) |  |  | **0.46** |  |
| **Dimension 4: Neurovegetative** |  |  |  |  |
| BDI19b: I have gained more than 15 pounds^b^ |  |  |  | **0.70** |
| BDI18b: I am eating a lot more than usual^b^ |  |  |  | **0.64** |
| BDI18c: I have had irresistible craving for sweets and starches lately^b^ | | |  | **0.49** |
| BDI19a: I have lost more than 15 pounds^b^ |  |  |  | **-0.40** |
| BDI17: I get too tired to do anything |  |  |  | **0.35** |
| **Remaining (unloaded) Items**  BAI16: Fear of dying  BDI16: I wake up early every day and can’t get more than 5 hours of sleep  BDI18a: I have no appetite at all anymore  BDI20: I am completely absorbed in what I feel/ am concerned about health  BDI21: I have lost interest in sex completely  BDI22: I have had at least 3-hours increase in sleep length^b^  DASS11: I found myself getting agitated (S)  DASS14: I was intolerant of anything that kept me from getting on with what I was doing (S)  DASS18: I felt that I was rather touchy (S) |  |  |  |  |

*Note.* Rotation: Promax; Items loadings below .35 were suppressed.

^a^DASS sub-scales, (D) represents depression, (A) represents anxiety and (S) represents stress items.

^b^additional BDI items.

**Supplementary** **Table** **S3.** **Percentage Change in Within-Cluster Heterogeneity Based on Number of Clusters Retained by Cluster Analysis**

| Stage | Number of clusters retained | Agglomeration coefficient | Percentage increase in heterogeneity to next stage |
| --- | --- | --- | --- |
| 529 | 10 | 576.85 | 6.08 |
| 530 | 9 | 611.90 | 6.34 |
| 531 | 8 | 650.71 | 8.22 |
| 532 | 7 | 704.19 | 7.97 |
| **533** | **6** | **760.32** | **10.51** |
| 534 | 5 | 840.24 | 12.21 |
| 535 | 4 | 942.82 | 12.66 |
| 536 | 3 | 1062.18 | 31.28 |
| 537 | 2 | 1394.45 | 54.36 |
| 538 | 1 | 2152.00 |  |

*Note.* Bolding indicates the stage at which clusters were determined to be retained.

**Supplementary Figure S1. Dendrogram for Hierarchical Clustering.*
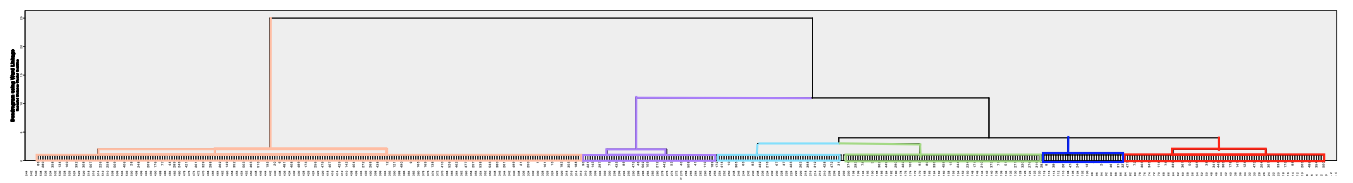
****Note.* Colours represent the six clusters chosen.
